# Accelerometer-derived physical activity in those with cardio-metabolic disease compared to healthy adults: a UK Biobank study of 52,556 participants

**DOI:** 10.1007/s00592-018-1161-8

**Published:** 2018-05-28

**Authors:** Sophie Cassidy, Harley Fuller, Josephine Chau, Michael Catt, Adrian Bauman, Michael I. Trenell

**Affiliations:** 1 Clinical Exercise Research Group, Institute of Cellular Medicine, Faculty of Medical Sciences, Newcastle University, 4th Floor William Leech Building, Newcastle upon Tyne, NE2 4HH UK; 2 Prevention Research Collaboration, Sydney School of Public Health, Charles Perkins Centre D17, Level 6 The Hub, University of Sydney, Sydney, NSW 2006 Australia; 3 Institute of Neuroscience, Faculty of Medical Sciences, Newcastle University, Newcastle upon Tyne, NE2 4HH UK; 4 NIHR Innovation Observatory, Newcastle University, Newcastle Upon Tyne, UK

**Keywords:** Accelerometer, Physical activity, Cardiovascular disease, Type 2 diabetes

## Abstract

**Aim:**

Cardio-metabolic disease and physical activity are closely related but large-scale objective studies which measure physical activity are lacking. Using the largest accelerometer cohort to date, we aimed to investigate whether there is an association between disease status and accelerometer variables after a 5-year follow-up.

**Methods:**

106,053 UK Biobank participants wore a wrist-worn GENEactiv monitor. Those with acceptable wear time (> 3 days) were split into 4 cardio-metabolic disease groups based on self-report disease status which was collected 5 ± 1 years prior. Multiple linear regression models were used to investigate associations, controlling for confounders and stratified for gender.

**Results:**

Average daily acceleration was lower in men (‘healthy’-42 ± 15 mg v ‘Type 2 diabetes + cardiovascular disease (CVD)’-31 ± 12 mg) and women (‘healthy’-44 ± 13 mg v ‘Type 2 diabetes + CVD’-31 ± 11 mg) with cardio-metabolic disease and this was consistent across both week and weekend days. Men and women with the worst cardio-metabolic disease perform around half of moderate to vigorous physical activity on a daily basis compared to healthy individuals, and spend almost 7 h per day in 30 min inactivity bouts. Significant associations were seen between cardio-metabolic disease and accelerometer variables 5 years on when controlling for confounders.

**Conclusion:**

In the largest accelerometer cohort to date, there are significant associations between cardio-metabolic disease and physical activity variables after 5 years of follow-up. Triaxial accelerometers provide enhanced measurement opportunities for measuring lifestyle behaviours in chronic disease.

## Introduction

Physical activity is closely associated with cardio-metabolic health [1], therefore accurate assessment is vital. Objective measures of physical activity have become widespread and offer numerous advantages over self-report methods. Over the past decade, there has been a shift in using hip-worn uniaxial accelerometers, to wrist-worn triaxial accelerometers which continuously sample and store raw acceleration. These offer improved precision and enhanced measurement opportunities, but large cohorts with triaxial accelerometer are lacking. Prior to the UK Biobank, the largest cohort of individuals with these devices included around 20,000 individuals [2]. This highlights the scale of the UK Biobank Study, whereby data was collected and analysed in > 100,000 participants—making it the world’s largest objective study of its kind. We used the UK Biobank, to investigate whether there is an association between cardio-metabolic disease status and accelerometer variables after a 5-year follow-up.

## Materials and methods

UK Biobank baseline assessments occurred between 2007 and 2010, when the following covariate data was collected; age, BMI, Townsend Deprivation Index, ethnicity, smoking and alcohol status, fruit and vegetable consumption, self-report weekly moderate to vigorous physical activity (MVPA). Self-report disease status was used to define four disease groups spanning cardio-metabolic health, which included ‘Healthy’ (individuals who reported no disease), ‘Cardiovascular disease (CVD)’, Type 2 diabetes minus CVD, and ‘Type 2 diabetes + CVD’.

Between February 2013 and December 2015, a subset of individuals was invited to wear an Axivity AX3 wrist-worn triaxial accelerometer for 7 consecutive days. Raw accelerometer data were processed using GGIR V1.5-9 package (R Core Team, Vienna, Austria) (https://cran.rroject.org/web/packages/GGIR/index.html) [3]. We defined MVPA using a 100 milligravity (m*g*) cut-off, based on laboratory findings [4]. Similarly, 6 metabolic equivalents (METs) is classified as vigorous activity and was equivalent to an acceleration around 400 mg [4]. ‘Light’ (< 3 METs) was defined as anything between 40 and 100 mg, and ‘Inactivity’ as anything below 40 mg [2]. Within each threshold, total activity time within waking hours was calculated (Light time, Moderate time, Vigorous time, Inactivity time). Additionally, time spent in 1–5 min (MVPA1min) and 10 min (MVPA10min) bouts of MVPA and time spent in 30 min of inactivity (Inactivity30mins) was calculated.

### Statistical analysis

Due to the large sample size, any small difference in acceleration mean was significant, therefore these results are not reported. Multiple linear regression models were used to investigate the association between cardio-metabolic disease and objective physical activity, after adjusting for baseline covariates. Physical activity variables did not meet assumptions of normality, therefore were transformed. All analyses were stratified by gender due to significant interaction between gender and outcome variables. To determine the robustness of the results, sensitivity analysis was performed with a more stringent cut-point of 120 and 50 mg to define MVPA and inactivity, respectively. Analyses were performed using SAS OnDemand for Academics (SAS Institute, Care, North Carolina, USA) software.

## Results

103,578 datasets were received, but only 52,556 fit into the four disease groups and were analysed. Those excluded reported a wide range of other diseases including respiratory, gastrointestinal, renal, neurology, musculoskeletal, haematology, gynaecology, immunological and infectious. Those with cardio-metabolic disease were less active during waking hours, demonstrated by a reduction in daytime acceleration (m*g*) in both men and women, which was consistent in both week and weekend days (Table 1). Even in the most active 5 h of the day, those with cardio-metabolic disease performed a lower intensity of activity compared to those with no disease. During waking hours, total time spent in each of the activity thresholds (light time, moderate time and vigorous time) declined across cardio-metabolic disease groups. Both men and women with ‘Type 2 diabetes + CVD’ performed half the level of MVPA when considered as MVPA1min or MVPA10min bouts. Inactive time was higher across all cardio-metabolic groups and a similar pattern was observed for Inactivity30min, whereby those with ‘Type 2 diabetes + CVD’ spent almost 7 h of the day in 30 min inactivity bouts (Inactivity30min).

**Table 1:** Physical activity acceleration values from Axivity in all participants (*n* = 52,424) according to disease status and stratified for gender

	Male (*n* = 24,880)				Female (*n* = 27,544)			
	Healthy (*n* = 11,232)	CVD (*n* = 11,996)	Type 2 diabetes minus CVD (*n* = 434)	Type 2 diabetes + CVD (*n* = 1218)	Healthy (*n* = 14,960)	CVD (*n* = 11,746)	Type 2 diabetes minus CVD (*n* = 277)	Type 2 diabetes + CVD (*n* = 561)
Age, years (SD)	54.3 (8.0)	59.6 (6.8)	59.1 (6.9)	61.0 (5.9)	53.6 (7.6)	58.5 (7.0)	58.6 (6.2)	59.9 (6.5)
BMI, kg/m^2^ (SD)	26.3 (3.5)	28.4 (4.2)	29.5 (4.4)	31.4 (5.3)	25.2 (4.1)	28.0 (5.4)	31.4 (6.5)	33.2 (6.4)
Physical activity								
Average acceleration values, m*g* (SD)								
Daytime acceleration	42 (15)	36 (12)	34 (11)	31 (12)	44 (13)	38 (12)	35 (12)	31 (11)
Acceleration for least active 5 h	0.63 (1.04)	0.69 (1.10)	0.69 (0.74)	0.79 (0.93)	0.54 (0.94)	0.57 (0.73)	0.70 (1.36)	0.72 (0.82)
Acceleration for most active 5 h	67 (28)	56 (22)	52 (18)	47 (25)	66 (23)	57 (18)	51 (18)	46 (17)
Weekday acceleration across night and day	30 (6)	26 (8)	24 (7)	22 (11)	30 (8)	27 (8)	24 (7)	22 (7)
Weekend acceleration across night and day	30 (12)	25 (10)	23 (8)	21 (7)	30 (10)	26 (8)	23 (8)	21 (7)
Total time spent across different thresholds during waking time (min/day)								
Inactivity time	588 (75)	604 (3)	615 (78)	624 (74)	568 (77)	583 (75)	599 (73)	617 (80)
Light time	162 (47)	156 (47)	153 (50)	146 (48)	182 (46)	178 (48)	167 (55)	156 (53)
Moderate time	96 (45)	79 (40)	72 (39)	61 (38)	104 (44)	87 (43)	75 (43)	62 (40)
Vigorous time	6.12 (7.6)	3.58 (5.04)	2.64 (3.01)	1.87 (2.50)	4.8 (6.3)	2.8 (3.8)	2.1 (3.3)	1.5 (2.5)
Bouts of activity during waking time (min/day)								
MVPA10min	22 (28)	14 (20)	13 (19)	8 (19)	20 (25)	12 (19)	9 (17)	5 (12)
MVPA1min	23 (14)	18 (12)	16 (12)	13 (11)	25 (14)	20 (13)	16 (13)	13 (12)
Inactivity30min	357 (124)	394 (128)	412 (134)	432 (133)	318 (115)	353 (122)	380 (139)	419 (141)

**Fig. 1:**
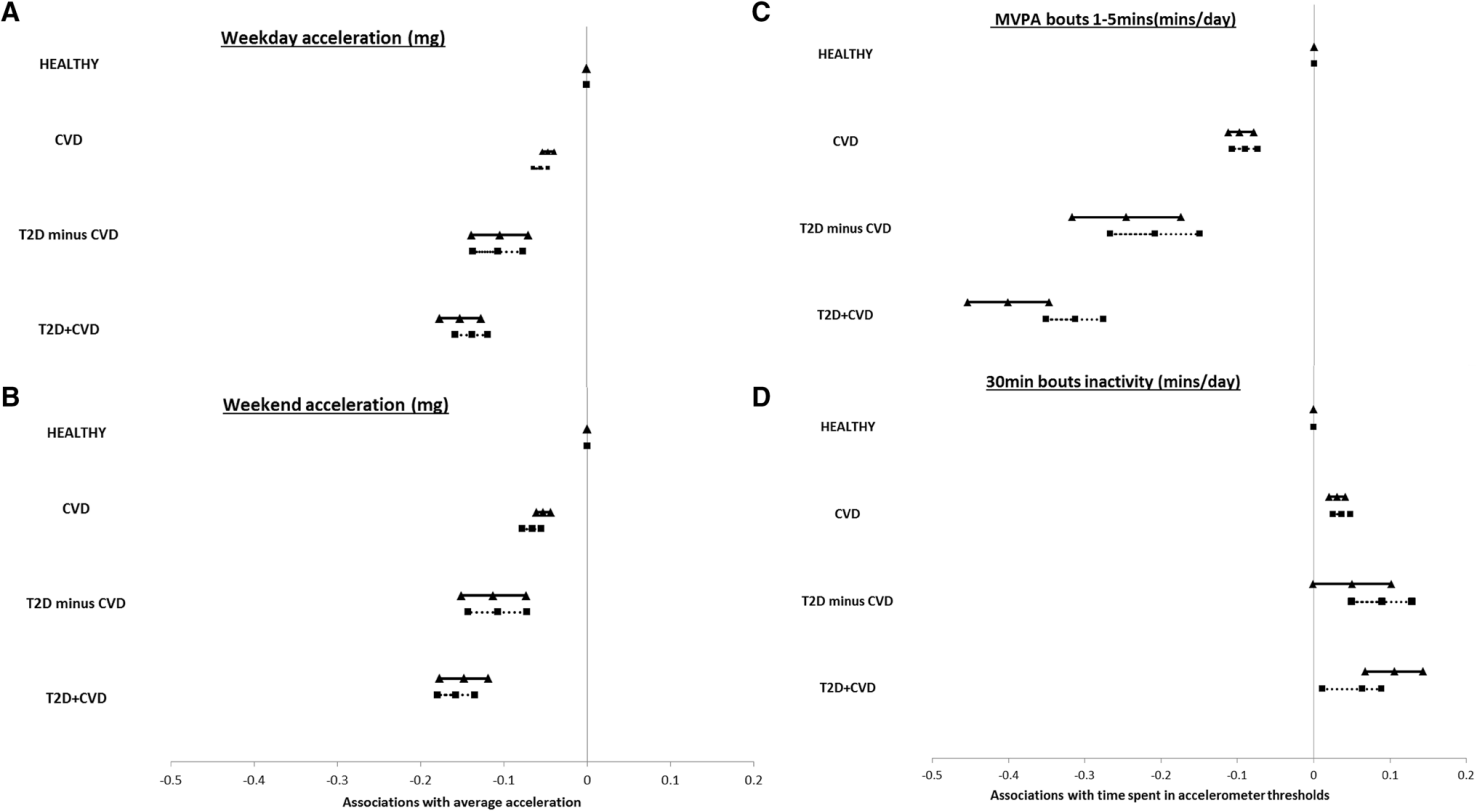
Associations of disease group at baseline with objective physical activity after an average 5 ± 1 years of follow-up. Weekday acceleration and Inactivity30min were log transformed, and weekend acceleration and MVPA1min were log+1 transformed. Models were adjusted for age, BMI, Townsend Deprivation Index, ethnicity, smoking, fruit and vegetable intake, alcohol, self-report weekly MVPA, follow-up time (Women = solid line + triangle, Men = dotted line + square). *T2D* type 2 diabetes

Figure 1 shows the prospective associations of baseline disease status with objective physical activity variables after 5 years of follow-up, when adjusting for confounders such as BMI. There were significant inverse associations of disease status with average acceleration across week and weekend days for both men and women. Similar patterns were seen with bouted and unbouted MVPA, whereas disease status demonstrated a slightly weaker positive association between bouted and unbouted inactivity. Sensitivity analysis demonstrated similar associations.

## Discussion

In the largest objective cohort to date, there is a decrease in objectively measured physical activity from healthy to CVD to diabetes patients which was consistent whether week or weekend acceleration was measured, or bouted or unbouted activity was used. These results demonstrate the usefulness of accelerometers in exploring the relationship between physical activity and chronic disease. The results also provide a platform for future novel explorations including the temporal distribution and patterns of physical activity, sleep and sedentary behaviour.

These devices make it possible to measure bouted and unbouted activity. We chose to focus on MVPA bouts in regression models, as it has been previously shown that bouts of MVPA have stronger associations with metabolic health, compared to unbouted activity [5]. Time spent in unbouted activity seems higher than what would be expected, with an average of around 60 min/day for those with ‘Type 2 diabetes and CVD’, but this could capture sporadic arm movements which cannot be separated from true physical activity. For this reason, identifying associations with > 1 min bouts of MVPA is the most informative. Inactivity levels were high regardless of disease status, however in this study, associations between inactivity and cardio-metabolic were not as strong as other accelerometer variables. This most likely reflects the methodological limitations in defining ‘inactivity’ using accelerometers. Due to the postural component, it is currently difficult to distinguish between light physical activity, e.g., standing, and sedentary behaviour (i.e., reclining/sitting), but efforts are underway to validate and define this behaviour using accelerometers, as well as sleep. An important limitation with this study is that the direction of causality cannot be distinguished.

Overall, strong and consistent relationships between cardio-metabolic disease and triaxial accelerometry, demonstrate enhanced measurement opportunities and greater insights going forward.

## Abbreviations

CVD: Cardiovascular disease
METs: Metabolic equivalents
*mg*: Milligravity
MVPA: Moderate to vigorous physical activity
